# DHA increases adiponectin expression more effectively than EPA at relative low concentrations by regulating PPARγ and its phosphorylation at Ser273 in 3T3-L1 adipocytes

**DOI:** 10.1186/s12986-017-0209-z

**Published:** 2017-08-11

**Authors:** Jia Song, Cheng Li, Yushan Lv, Yi Zhang, William Kwame Amakye, Limei Mao

**Affiliations:** Department of Nutrition and Food Hygiene, Guangdong Provincial Key Laboratory of Tropical Disease Research, School of Public Health, Southern Medical University, Guangzhou, Guangdong China

**Keywords:** Adiponectin, DHA, EPA, PPARγ, PPARγ phosphorylation

## Abstract

**Background:**

Enhancing circulating adiponectin is considered as a potential approach for the prevention and treatment of non-communicable diseases (NCDs). Docosahexaenoic acid (DHA) and eicosapentaenoic acid (EPA) were reported to increase adiponectin by previous studies using a mixture of them. However, their individual effects on adiponectin and the underlying mechanisms are still unclear. In the present study, we observed and compared the individual effect of DHA and EPA on adiponectin in 3T3-L1 adipocytes, and further tested whether DHA or EPA regulated adiponectin by peroxisome proliferator-activated receptor γ (PPARγ) and its phosphorylation at Ser273 to provide a plausible explanation for their distinct actions.

**Methods:**

Firstly, 3T3-L1 adipocytes were treated with different doses of DHA or EPA for 24 h. Secondly, 3T3-L1 adipocytes were treated with DHA or EPA in the presence or absence of GW9662. Thirdly, 3T3-L1 adipocytes were pretreated with DHA or EPA for 24 h, followed by being respectively co-incubated with tumor necrosis factor α (TNF-α) or roscovitine for another 2 h. Bovine serum albumin treatment served as the control. After treatments, cellular and secreted adiponectin, cellular PPARγ and its phosphorylation at Ser273 were determined.

**Results:**

Compared with the control, DHA increased cellular and secreted adiponectin at 50 and 100 μmol/L, while EPA increased them at 100 and 200 μmol/L. Adiponectin expressions in DHA treated groups were significantly higher than those in EPA treated groups at 50 and 100 μmol/L. Both DHA and EPA enhanced PPARγ expression, but DHA was more effective. GW9662 blocked DHA- and EPA-induced increases in PPARγ as well as adiponectin. Remarkably, an opposite regulation of PPARγ phosphorylation was detected after fatty acids treatment: DHA inhibited it but EPA stimulated it. TNF-α blocked DHA-induced decrease in PPARγ phosphorylation, which eventually led to a decrease in adiponectin. Roscovitine blocked EPA-induced increase in PPARγ phosphorylation, but the corresponding increase in adiponectin was non-significant.

**Conclusion:**

DHA compared with EPA led to a greater increase in cellular and secreted adiponectin at relative low concentrations by increasing PPARγ expression and inhibiting its phosphorylation at Ser273. DHA may be more beneficial than EPA in reducing risks of NCDs.

## Background

In recent decades, the rapid rise in major non-communicable diseases (NCDs) such as diabetes mellitus, cardiovascular disease and cancer has delivered a great threat to public health worldwide. Adipose tissue dysfunction, characterized by adipocytes hypertrophy, mitochondrial dysfunction and abnormal secretion of adipokines and cytokines, is closely related to the pathogenesis of metabolic disorders, which eventually contribute to the development and progression of NCDs [1–3]. Adiponectin is an adipocyte-derived adipokine with well documented insulin-sensitizing, anti-inflammatory and anti-atherogenic properties [4]. As a significant change of adipose tissue dysfunction, a decreased level of adiponectin has been identified as an independent risk factor for NCDs by a growing body of clinical research [5, 6]. Modulation of adiponectin to a higher level is therefore considered to be a potential approach for the prevention and treatment of NCDs.

Numerous nutritional factors are reported to be associated with the regulation of adiponectin expression like carbohydrate, vegan protein and fatty acids [7–9], among which omega-3 polyunsaturated fatty acid (PUFA) especially eicosapentaenoic acid (EPA) and docosahexaenoic acid (DHA) were shown to significantly increase circulating concentration of plasma adiponectin in humans [10–12]. However, inconsistent results were obtained from several randomized controlled trials (RCTs) showing EPA and DHA supplementation did not change plasma adiponectin levels [13, 14]. The discrepancy is possibly due, in part, to differences in demographic characteristic of subjects, dose of EPA and DHA and duration of intervention. Remarkably, individual effect of EPA and DHA is proposed to be another important reason since recent RCTs reported DHA-enriched canola oil obviously increased plasma adiponectin level in adults [15] whereas ethyl-EPA failed to demonstrate similar effect [16]. These evidences suggest that different effects may exist between DHA and EPA with respect to the modulation of adiponectin expression. Unfortunately, limited research is specifically designed to explore whether DHA and EPA have equivalent or distinct biological actions.

Understanding the molecular mechanism by which omega-3 PUFA regulates adiponectin expression is helpful to explain the potential differences between DHA and EPA. However, the mechanism is still incompletely clarified. Nuclear peroxisome proliferator-activated receptor γ (PPARγ) has been recognized as a critical regulator of adiponectin gene transcription [17]. DHA and EPA were demonstrated to stimulate adiponectin expression by activating PPARγ in vitro [18, 19], but this result is controversial [20]. The definite relationship between PPARγ and adiponectin under DHA or EPA treatment is still unclear. Besides, the phosphorylation of PPARγ at serine (Ser) 273 mediated by cyclin dependent kinase 5 (CDK5) proposed by Choi et al. [21] may be another important mechanism for the modulation of adiponectin expression. An increase in PPARγ phosphorylation at Ser273 contributed to a marked reduction of adiponectin in vitro and in vivo. Insulin sensitizer rosiglitazone (a synthesized PPARγ agonist) could increase adiponectin expression by blocking CDK5-mediated PPARγ phosphorylation [21]. Being natural ligands for PPARγ [22], DHA and EPA are hypothesized to work in a similar way, but conclusive evidence is still lacking.

In the present study, the individual effect of DHA and EPA on adiponectin expression was compared primarily after treating 3T3-L1 adipocytes with each at different concentrations. Secondly, whether DHA or EPA regulated adiponectin by PPARγ and its phosphorylation at Ser273 was tested to provide a plausible explanation for their distinct actions. The final results will be helpful in developing an efficient nutritional strategy to improve adiponectin and reduce the risk of NCDs.

## Methods

### Cell culture and differentiation

**Fig. 1:**
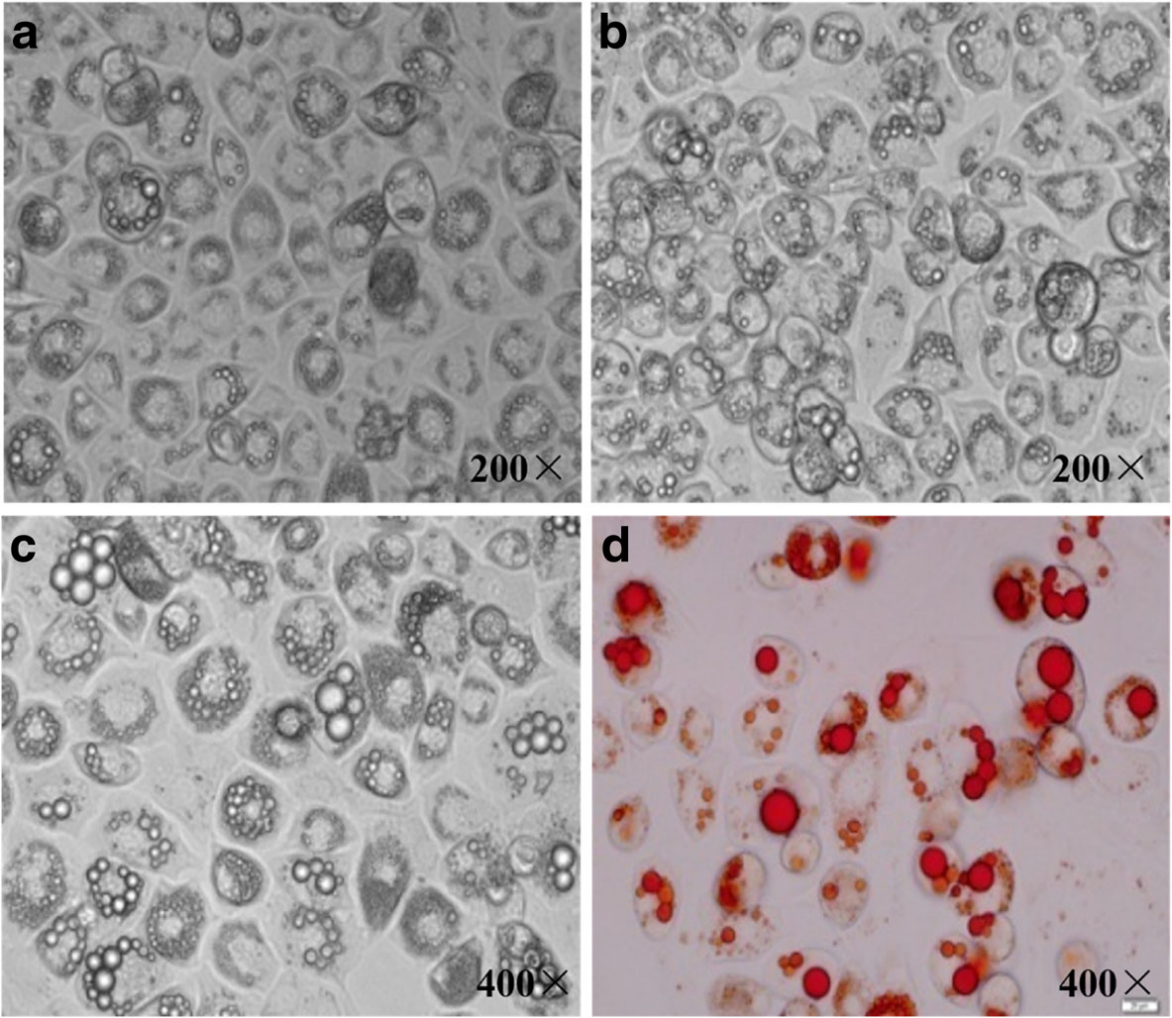
Differentiation of 3T3-LI adipocytes. 3T3-L1 adipocytes at day 4 (**a**), 6 (**b**) and 8 (**c**) after differentiation. Mature 3T3-L1 adipocytes were stained by Oil *Red* O (**d**)

The 3T3-L1 mouse embryo fibroblasts (termed 3T3-L1 preadipocytes) were purchased from American Type Culture Collection (Manassas, VA, USA). 3T3-L1 preadipocytes were maintained in Dulbecco’s Modified Eagle’s Medium (DMEM, Gibco, Carlsbad, CA, USA) supplemented with 10% fetal bovine serum (ExCell, Shanghai, CHN) and 1% antibiotic (10,000 U/mL penicillin and 10,000 U/mL streptomycin, PanEra, Guangzhou, CHN) at 37 °C in 5% CO_2_ in a humidified incubator. After becoming completely confluent, 3T3-L1 preadipocytes were stimulated to differentiate in the above growth medium containing 10 μg/mL insulin, 0.5 mmol/L 3-isobutyl-1-methyl-xanthine (IBMX) and 1 μmol/L dexamethasone (all from Sigma, St. Louis, MO, USA) for 2 days, followed by exposing to the growth medium only containing 10 μg/mL insulin for 2 more days. At day 4, cells were maintained in growth medium again for 4 to 6 days until more than 85% of them were filled with lipid droplets. Mature 3T3-L1 adipocytes were identified by Oil Red O staining (Fig. 1).

### Fatty acids treatments

Purified DHA and EPA (Sigma, St. Louis, MO, USA) were dissolved in ethanol and combined with 10% fatty acid-free bovine serum albumin (BSA, Sigma, St. Louis, MO, USA) at a molar ratio of 1:2 to prepare fatty acid-BSA stock solutions. After a 12-h serum starvation, 3T3-L1 adipocytes were treated with DHA- or EPA-BSA stock solutions which were previously diluted in serum-free growth medium (DMEM only supplemented with 1% antibiotic) for 24 h. The final concentrations of DHA and EPA obtained were 25, 50, 100 and 200 μmol/L. BSA treatment served as the control.

### MTT assay

Cell viability was tested according to the protocol modified from Rhyu et al. [23]. 3T3-L1 preadipocytes were seeded in a 96-well microculture plate at a concentration of 1 × 10^4^ cells/mL. Post-confluent 3T3-L1 preadipocytes were treated with different doses (25, 50, 100 and 200 μmol/L) of DHA or EPA for 24 h, while BSA treatment served as the control. Approximately 20 μL of 5 mg/mL 3-(4, 5-dimethylthiazol-2-yl)-2, 5-diphenyl tetrazolium bromide (MTT, Sigma, St. Louis, MO, USA) was added to each of the above treatment wells for 4 h in an incubator. Then the medium was removed and 150 μL of dimethylsulfoxide (DMSO, Sigma, St. Louis, MO, USA) was added into each well to dissolve the formazan crystals. Optical density (OD) values were measured at 570 nm by Epoch2 microplate reader (Biotek, Winooski, VT, USA) and normalized to the percentage of control.

### Lipid peroxidation

After finishing DHA or EPA treatments at various concentrations, culture media were collected and centrifuged at 3000 rpm for 10 min. The concentrations of malondialdehyde (MDA) and total superoxide dismutase (SOD) in the supernatants were determined by thibabituric acid method and hydroxylamine method respectively, according to the manufacturer’s instructions of commercial kits purchased from Nanjing Jiancheng Bioengineering Institute, Jiangsu, China.

### GW9662 treatment

GW9962 (a PPARγ antagonist, Sigma, St. Louis, MO, USA) was dissolved in DMSO to prepare the stock solution. After a 12-h serum starvation, 3T3-L1 adipocytes were treated with 100 μmol/L DHA or EPA (chosen by does-dependent effect of fatty acid on cellular adiponectin expression, as shown in Fig. 3) in the presence or absence of 10 μmol/L GW9662, or GW9662 alone for 24 h.

### TNF-α and roscovitine treatments

TNF-α (PeproTech, Rocky Hill, NJ, USA) and roscovitine (Sigma, St. Louis, MO, USA) were used to regulate the phosphorylation of PPARγ at 273 Ser by activating or inhibiting CDK5 activity [21]. Both of them were dissolved in DMSO to prepare the stock solutions. After a 12-h serum starvation, 3T3-L1 adipocytes were pretreated with 100 μmol/L DHA or EPA for 24 h, followed by adding 20 ng/mL TNF-α or 10 μmol/L roscovitine to the medium for another 2 h (chosen through the pilot works, as shown in Fig. 5b), or treated with TNF-α or roscovitine alone for 2 h.

### RNA isolation and real-time PCR

Total RNA was extracted using TRIzol (Invitrogen, Carlsbad, CA, USA) and quantified by Nanodrop 2000 (Thermo Fisher, Waltham, MA, USA). Approximately 500 ng of total RNA were reverse transcribed to cDNA in a 10 μL of reaction mixture using PrimeScript™ RT reagent kit (Takara, Dalian, CHN) and thermal cycler S1000 (Bio-Rad, Hercules, CA, USA). Then, cDNA (100 ng) was amplified by real-time PCR in a 25 μL of reaction mixture to determine the relative expressions of adiponectin and PPARγ mRNA. House keeper gene β-actin was amplified in parallel as the internal reference. Real-time PCR was performed using SYBR premix Ex Taq II (Takara, Dalian, CHN) and Mx3005P qPCR system (Agilent, PaloAlto, FL, USA). The following primers were used: 5′-GTGGGAATGGGTCAGAAGGA-3′ (forward) and 5′-CTTCTCCATGTCGTCCCAGT-3′ (reverse) for β-actin; 5′-TACTGCAACATTCCGGGACT-3′ (forward) and 5′-GAACGGCCTTGTCCTTCTTG-3′ (reverse) for adiponectin; 5′-AACTCCCTCATGGCCATTGA-3′ (forward) and 5′-CCTTGCATCCTTCACAAGCA-3′ (reverse) for PPARγ. Amplification procedure of real-time PCR was 1 cycle of 95 °C for 30 s, followed by 40 cycles of 95 °C for 5 s and 60 °C for 30 s. Relative expressions of target genes were calculated using 2^-△△Ct^ method [24].

### Protein isolation and western blot

Total cellular proteins were extracted using ice cooled strong RIPA lysis buffer containing 1 mmol/L phenylmethanesulfonyl fluoride and 1 mmol/L phosphatase inhibitor cocktails (all from KeyGEN BitoTECH, Nanjing, CHN), and quantified by bicinchoninic acid protein assay kit (ExCell, Shanghai, CHN). Mixtures of cellular proteins and sodium dodecyl sulfate polyacrylamide gel electrophoresis (SDS-PAGE) loading buffer (5×, PanEra, Guangzhou, CHN) were heated at 100 °C for 10 min. Approximately 40 μg of denatured proteins were loaded and separated by SDS-PAGE (12% acrylamide), and then transferred to the polyvinylidene difluoride membranes (0.45 μm, Millipore, Bedford, MA, USA) using a wet-transfer system at 100 V for 50 min. After blocking with 5% nonfat milk which was dissolved in Tris-buffered saline-Tween (TBST, 0.1% Tween), membranes were separately incubated with adiponectin-specific (1:500 dilution), PPARγ-specific (1:1000) and β-actin-specific primary antibodies (1:1000, all from Santa Cruz, CA, USA) overnight at 4 °C. Membranes were thereafter rinsed five times with TBST washing solution, followed by incubating with corresponding horseradish peroxidase co-conjugated secondary antibodies (1:2000 dilution for anti-mouse IgG and 1:5000dilution for anti-rabbit IgG, all from Santa Cruz, CA, USA) for 1.5 h at room temperature. After washing, strips in membranes were visualized using chemiluminescent peroxidase substrate (Millipore, Bedford, MA, USA) and Tanon-5200 chemical luminescence developing system (Tanon, Shanghai, CHN). β-actin served as the internal reference. The relative expressions of cellular adiponectin and PPARγ proteins in treatment groups were determined by grey value analysis using Image J software (Bethesda, MD, USA), and normalized to the control.

### Adiponectin secretion

Cell culture media were collected and centrifuged for 10 min at 5000 r/min to acquire culture supernates. Concentrations of secreted adiponectin in culture supernates were quantified by a commercial mouse adiponectin ELISA kit (R and D, Minneapolis, MN, USA). Measurements were repeated three times.

### Co-immunoprecipitation assay

Quantification of phosphorylation of PPARγ at 273 Ser was performed by co-immunoprecipitation method as previously described [21]. Approximately 500 μg of total cellular proteins were pre-treated with 1 μg of PPARγ-specific primary antibody for 2 h at 4 °C, and then co-incubated with 20 μL of protein A/G plus-agarose (Santa Cruz, CA, USA) overnight at 4 °C. Mixtures of proteins, antibody and agarose were centrifuged at 2500 rpm for 5 min at 4 °C to collect immunoprecipitates. The pellets were washed four times with RIPA lysis buffer and suspended by 20 μL of 2× SDS-PAGE loading buffer. After heat-denaturation samples were analyzed by SDS-PAGE method as above (part 2.8) using 5% BSA dissolved in TBST for blocking, 1:1000 diluted phosphor-CDK substrate motif [(K/H) pSP] primary antibody (CST, Boston, MA, USA), 1:500 diluted PPARγ-specific primary antibody and corresponding secondary antibodies for incubation. The ratios of p-PPARγ to PPARγ in treatment groups were calculated and normalized to the control to represent the relative concentrations of phosphorylation of PPARγ at Ser273.

### Statistical analysis

All the experiments were separately done at least three times. Data were presented as means ± standard deviations (SD) and analyzed by SPSS 20 software (SPSS, Chicago, IL, USA). Student’s *t* test was used to identify differences between two independent groups. Multiple group differences were analyzed using One-way analysis of variance (ANOVA) followed by Student-Newman-Keuls (SNK) test. Statistical significance was set at *P* < 0.05.

## Results

### Effects of DHA and EPA on cell viability and lipid peroxidation indexes

**Fig. 2:**
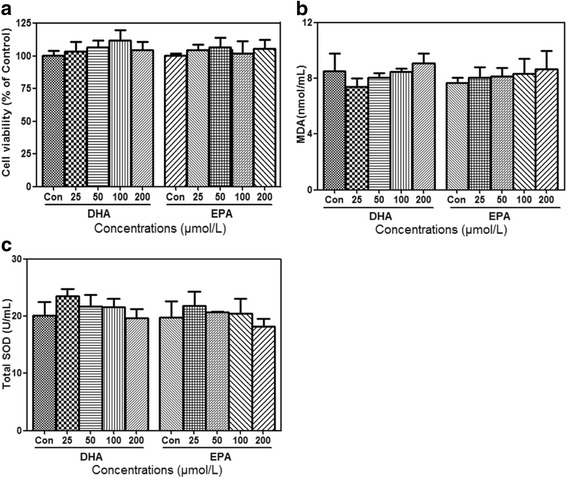
Effects of DHA and EPA on cell viability and lipid peroxidation indexes. Post-confluent 3T3-L1 preadipocytes and 3T3-L1 adipocytes were separately treated with different doses (25, 50, 100 and 200 μmol/L) of DHA or EPA for 24 h, while BSA treatment served as the control. Cell viabilities (**a**) of preadipocytes in treatment groups were assessed and normalized to the percentage of control. MDA (**b**) and total SOD (**c**) levels in culture media of adipocytes were measured to represent the risk of lipid peroxidation. Data were presented as mean ± SD, *n* = 3. One-way ANOVA followed by Student-Newman-Keuls (SNK) test. Con: control

Post-confluent 3T3-L1 preadipocytes and adipocytes were separately treated with different doses (25, 50, 100 and 200 μmol/L) of DHA or EPA for 24 h. Results of MTT assay demonstrated that neither DHA nor EPA changed cell viabilities of preadipocytes (Fig. 2a). Since n-3 PUFAs are prone to lipid peroxidation [25], MDA and total SOD in culture media of adipocytes were measured after fatty acids treatment. As shown in Fig. 2b and c, both DHA and EPA had no significant effects on MDA and total SOD levels. Taken together, DHA and EPA did not lead to cytotoxicity and lipid peroxidation, suggesting the following results of the present study would not be affected by the potential adverse side effects of DHA and EPA.

### DHA increased cellular and secreted adiponectin more effectively than EPA at relative low concentrations

**Fig. 3:**
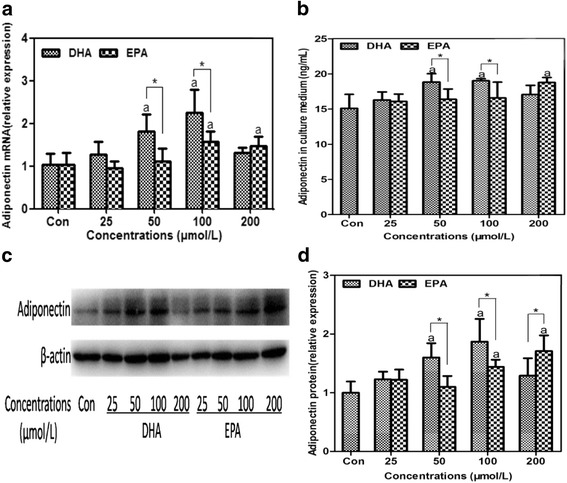
Dose-dependent effects of DHA and EPA on adiponectin synthesis and secretion. 3T3-L1 adipocytes were incubated with different doses (25, 50, 100 and 200 μmol/L) of DHA or EPA for 24 h, while BSA treatment served as the control. Cellular adiponectin (**a**, **b**, **c**) and secreted adiponectin (**d**) were assessed. Cellular adiponectin in treatment groups were normalized to the control with β-actin worked as the internal reference. Data were presented as mean ± SD, *n* = 4. ^a^ *P* < 0.05 versus control; ^*^ *P* < 0.05 DHA versus EPA. One-way ANOVA followed by Student-Newman-Keuls (SNK) test and student’s *t* test. Con: control

3T3-L1 adipocytes were treated with 25, 50, 100 and 200 μmol/L of DHA or EPA for 24 h to observe dose-dependent effects of fatty acids on adiponectin expression (Fig.3). DHA increased the cellular and secreted adiponectin to a greater extent as compared to the control (*P* < 0.05) at the concentrations of 50 and 100 μmol/L, and the most obvious changes were observed at 100 μmol/L. EPA exhibited similar effects on adiponectin at the concentrations of 100 and 200 μmol/L with the greatest changes observed at 200 μmol/L (*P* < 0.05). It was noteworthy that cellular and secreted adiponectin in DHA treated groups were significantly higher than those in EPA treated groups at 50 and 100 μmol/L (*P* < 0.05), whereas adiponectin protein in DHA treated group was lower than that in EPA treated group at 200 μmol/L (*P* < 0.05). As a result, DHA was more pronounced than EPA in stimulating adiponectin synthesis and secretion at relative low concentrations (50-100 μmol/L).

### DHA compared with EPA led to a greater increase in PPARγ

**Fig. 4:**
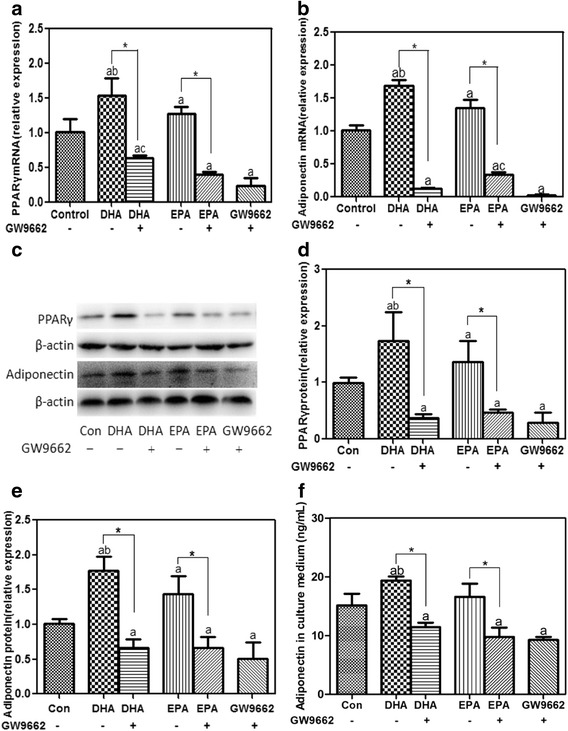
GW9662 blocked DHA- and EPA-induced increases in cellular and secreted adiponectin by inhibiting PPARγ. 3T3-L1 adipocytes were incubated with 100 μmol/L of DHA (or EPA) in the presence or absence of 10 μmol/L of GW9662, or GW9662 alone for 24 h. BSA treatment served as the control. Cellular PPARγ (**a**, **c**, **d**), adiponectin (**b**, **c**, **e**) and secreted adiponectin (**f**) were assessed. Cellular PPARγ and adiponectin in treatment groups were normalized to the control with β-actin worked as the internal reference. Data were presented as mean ± SD, *n* = 5. ^a^ *P* < 0.05 versus control; ^b^ *P* < 0.05 versus EPA; ^c^ *P* < 0.05 versus GW9662; ^*^ *P* < 0.05 DHA versus DHA + GW9662 and EPA versus EPA + GW9662. One-way ANOVA followed by Student-Newman-Keuls (SNK) test. Con: control

As natural ligands for PPARγ, DHA and EPA are supposed to increase adiponectin in a PPARγ-dependent manner. The present study treated 3T3-L1 adipocytes with 100 μmol/L of DHA or EPA for 24 h to observe the changes of PPARγ. Dose of DHA or EPA was chosen in accordance with their distinct dose-dependent effects on adiponectin. After treatment, both DHA and EPA triggered remarkable increases in cellular mRNA (Fig. 4a) and protein (Fig. 4c and d) expressions of PPARγ (*P* < 0.05). However, the increases in PPARγ mRNA and protein for DHA compared with EPA were significantly different (*P* < 0.05). DHA was more potent than EPA in simulating PPARγ expression.

### GW9662 blocked DHA- and EPA-induced increases in adiponectin by inhibiting PPARγ

To verify whether DHA or EPA regulated cellular and secreted adiponectin through PPARγ, 3T3-L1 adipocytes were incubated with DHA or EPA in the presence or absence of GW9662 (a PPARγ antagonist), or GW9662 alone for 24 h. As shown in Fig. 4, comparing to the control, GW9662 alone obviously inhibited mRNA and protein expressions of PPARγ (*P* < 0.05) with concurrent decreases in cellular and secreted adiponectin (*P* < 0.05). Meanwhile, PPARγ and adiponectin in adipocytes treated by DHA or EPA in combination with GW9662 were significantly lower than those in the control (*P* < 0.05). GW9662 obviously attenuated DHA- and EPA-induced increases in PPARγ as well as adiponectin (*P* < 0.05).

### Opposite effects of DHA and EPA on phosphorylation of PPARγ at Ser273

**Fig. 5:**
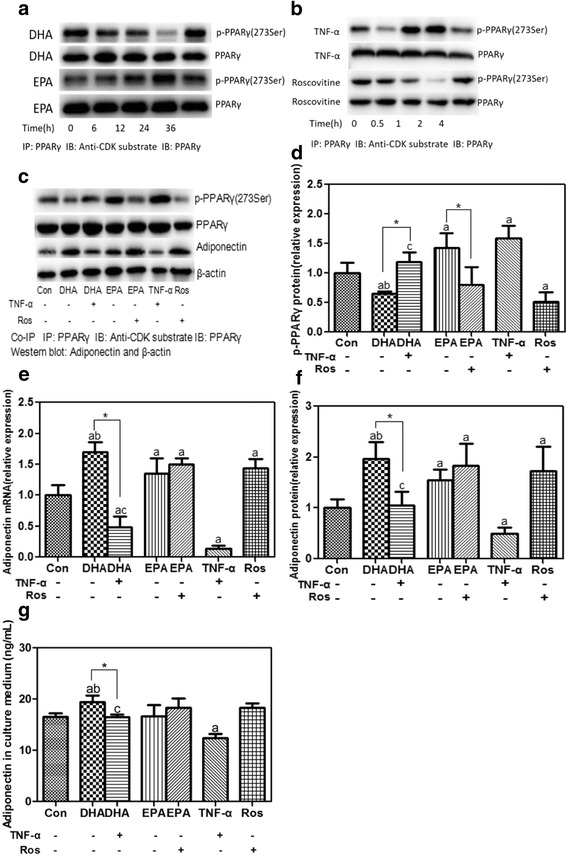
Role of PPARγ phosphorylation at Ser273 in DHA- or EPA-induced increases in adiponectin. **a** time-dependent effects of DHA and EPA (100 μmol/L) on phosphorylation of PPARγ at Ser 273. **b** time-dependent effect of TNF-α (20 ng/mL) or roscovitine (10 μmol/L) on phosphorylation of PPARγ at Ser 273. 3T3-L1 adipocytes were pretreated with 100 μmol/L of DHA or EPA for 24 h, followed by being respectively co-incubated with TNF-α or roscovitine for another 2 h. Besides, adipocytes were also incubated with DHA (24 h), EPA (24 h), TNF-α (2 h) or roscovitine (2 h) alone. BSA treatment served as the control. Cellular phosphorylation of PPARγ at Ser 273 (**c**, **d**) was assessed using co-immunoprecipitation assay and normalized to the control with PPARγ worked as the reference. Cellular adiponectin (**c**, **e**, **f**) and secreted adiponectin (**g**) were also quantified. Cellular adiponectin in treatment groups were normalized to the control with β-actin worked as the internal reference. Data were presented as mean ± SD, *n* = 5. ^a^ *P* < 0.05 versus control; ^b^ *P* < 0.05 versus EPA; ^c^ *P* < 0.05 versus TNF-α; ^*^ *P* < 0.05 DHA versus DHA + TNF-α and EPA versus EHA + roscovitine. One-way ANOVA followed by Student-Newman-Keuls (SNK) test. Co-IP: co-immunoprecipitation; Con: control; Ros: roscovitine

CDK5-induced phosphorylation of PPARγ at Ser273 reported by Choi et al. [21] may also play an important role in the regulation of adiponectin. Time-dependent effects of DHA and EPA (100 μmol/L) treatment demonstrated that the most obvious changes in phosphorylation of PPARγ at Ser273 were observed at 24 h (Fig. 5a). DHA treatment elicited a significant decrease in phosphorylation of PPARγ at Ser273 (*P* < 0.05), whereas EPA promoted it (*P* < 0.05, Fig. 5c and d) at 24 h. Obviously, DHA and EPA exerted an opposite effect on the regulation of phosphorylation of PPARγ at Ser273.

### Phosphorylation of PPARγ at Ser273 played an important role in DHA- and EPA-induced increases in adiponectin

Time-dependent effects indicated that the best time for TNF-α (a CDK5 agonist) and roscovitine (a CDK5 antagonist) to regulate the phosphorylation of PPARγ at Ser273 was 2 h (Fig. 5b). To elucidate whether DHA or EPA regulated cellular and secreted adiponectin through a modulation of phosphorylation of PPARγ at Ser273, 3T3-L1 adipocytes were pretreated with DHA or EPA for 24 h, followed by being respectively co-incubated with TNF-α or roscovitine for another 2 h. At the same time, 3T3-L1 adipocytes were also treated with DHA, EPA, TNF-α or roscovitine alone. As shown in Fig. 5c-g, TNF-α significantly promoted phosphorylation of PPARγ (*P* < 0.05) with concurrent decreases in cellular and secreted adiponectin (*P* < 0.05). What was more, the addition of TNF-α effectively blocked DHA-induced decrease in phosphorylation of PPARγ at Ser273 (*P* < 0.05), which finally led to a decrease in adiponectin (*P* < 0.05). Differing from TNF-α, roscovitine significantly increased cellular adiponectin expression by inhibiting the phosphorylation of PPARγ. EPA-induced increase in phosphorylation of PPARγ at Ser273 could be attenuated by roscovitine (*P* **<** 0.05), but the corresponding increase in adiponectin was minimal (*P* > 0.05).

## Discussion

A decreased level of adiponectin caused by adipose tissue dysfunction has been shown to be implicated in the development and progression of NCDs [26, 27]. DHA and EPA are suggested as potential inducers of adiponectin [18, 19], but there is limited information available regarding the definite individual effects of DHA and EPA on adiponectin and the underlying mechanisms, and results from existing experiments have always been controversial [14, 28, 29]. In the present study, DHA was proven to be more potent than EPA in stimulating adiponectin synthesis and secretion at relative low concentrations in 3T3-L1 adipocytes. Meanwhile, our result is the first to indicate that different magnitudes of increases in PPARγ expression and opposite modulations of PPARγ phosphorylation (DHA inhibited while EPA stimulated) are partly linked to the distinct impacts of DHA and EPA on adiponectin.

A concentration-dependent relationship between n-3PUFA and adiponectin indicated by a previous review [30] was also observed in the present study. As compared to the control, both DHA and EPA significantly increased cellular and secreted adiponectin in certain ranges of doses (DHA 50-100 μmol/L; EPA 100-200 μmol/L), and the best beneficial doses of DHA and EPA were 100 and 200 μmol/L, respectively. However, these results were inconsistent with the findings obtained by Romacho et al. [31] demonstrating that no significant alteration of cellular adiponectin was detected in adipocytes after a 24-h exposure to 100 μmol/L of DHA or EPA. It was previously reported that the effects of DHA and EPA on adiponectin depended on the stage of adipocyte maturation [32], so the above discrepancy can be partially explained by differences in magnitude of cellular maturation: old stage of maturation (14 days after differentiation) in Romacho et al. versus early stage of maturation (8 to 10 days after differentiation) in the present study.

Although both DHA and EPA stimulated adiponectin synthesis and secretion at specific concentrations, the optimal doses of fatty acids and magnitudes of increase in adiponectin were remarkably different. DHA was found to be more pronounced than EPA in enhancing cellular and secreted adiponectin levels at 50 and 100 μmol/L. A similar result was also observed in a recent RCT indicating that DHA supplementation compared with EPA supplementation led to a greater increase in plasma adiponectin in adults [33]. However, it should be noted that comparison of DHA and EPA in the present study also indicated that EPA induced a greater increase in cellular adiponectin protein than DHA at 200 μmol/L, which meant that differences between DHA and EPA may depend on concentration. At relative low concentrations (50-100 μmol/L), DHA was more effective than EPA in inducing adiponectin.

Being a member of the nuclear receptor super-family, PPARγ has been identified as a critical regulator of adipogenesis, glucose and lipid metabolism, insulin sensitivity and inflammation [34, 35]. A direct binding of PPARγ/retinoid X receptor heterodimer to a functional PPARγ response element, which is located in the promoter site of adiponectin gene, has been proven to effectively augment adiponectin gene transcription [17]. Besides, PPARγ was also reported to stimulate the translation [36] and secretion [37] of adiponectin protein through other signaling pathways in animals and cells. However, the role of PPARγ in n-3PUFA-mediated regulation of adiponectin is still undefined.

Results observed in the present study indicated that both DHA and EPA significantly increased the synthesis of PPARγ. The addition of GW9662, a classical PPARγ antagonist, drastically blocked DHA- and EPA-induced increases in PPARγ and adiponectin. These findings were similar to a previous study [18] but differed from results obtained by Oster RT et al. [38] in which EPA mediated increase in secreted adiponectin could not be blocked by BADGE (another PPARγ antagonist). Distinct kinds of PPARγ antagonists, GW9662 in our study versus BADGE in Oster RT et al., may in part be responsible for the discrepancy. Besides, it should be noted that Oster RT et al. did not explore the influences of BADGE on cellular PPARγ and adiponectin expressions, making it difficult to determine whether EPA regulated adiponectin in a PPARγ-dependent manner, and this has been identified as a shortcoming in that paper [38]. In the present study, the impact of GW9662 on PPARγ, cellular and secreted adiponectin were systematically explored. Our results provided convincing data suggesting that both DHA and EPA modulated adiponectin expression through PPARγ.

Increases in PPARγ posttranslational phosphorylation at serine residues induced by various kinases such as CDK5, extracellular signal-regulated kinase-1/2 and c-Jun N-terminal kinase were reported to be involved in the pathogenesis of insulin resistance, inflammation and obesity [21, 39, 40]. Choi et al. [21] found that the anti-diabetic function of rosiglitazone (a synthetic PPARγ ligand) was partially due to its stimulation in circulating adiponectin by inhibiting CDK5 mediated phosphorylation of PPARγ at Ser273. In analogy to rosiglitazone, DHA was shown to significantly decrease the phosphorylation of PPARγ at Ser273 by the present research. TNF-α could block DHA-induced increases in adiponectin synthesis and secretion by enhancing PPARγ phosphorylation. Unexpectedly, an increase in PPARγ phosphorylation was detected after EPA treatment. Available data demonstrated that PPARγ phosphorylation contributed to its degradation through the ubiquitin-proteasome system [41], which may eventually lead to a decrease in adiponectin, but we found EPA still increased PPARγ expression as well as adiponectin. It is therefore hypothesized that a direct up-regulation of PPARγ expression could effectively counteract the negative effect caused by an increase in PPARγ phosphorylation under EPA treatment.

One of the most important findings in the present study was that DHA compared with EPA led to greater increases in cellular and secreted adiponectin at relative low concentrations (50 and 100 μmol/L). Different modulations of DHA and EPA on PPARγ provide a plausible explanation for their distinct effects on adiponectin. Firstly, in accordance with an earlier research conducted by Murali G et al. [42], DHA was more potent than EPA in stimulating cellular PPARγ expression. A stronger modulation of PPARγ was proposed to be linked to a greater increase in adiponectin. Secondly, DHA inhibited PPARγ phosphorylation while EPA stimulated it. PPARγ phosphorylation was claimed to degrade PPARγ [41], as a result, EPA-induced increase in PPARγ phosphorylation possibly resulted in the lower increase in PPARγ expression as well as adiponectin as compared with DHA.

Currently, strategy aimed at increasing circulating adiponectin is considered as a potential approach for the prevention and treatment of obesity-related NCDs especially type 2 diabetes. As a kind of synthetic PPARγ agonist, anti-diabetic drug thiazolidinedione (TZD) has been shown to improve insulin sensitivity by enhancing adiponectin expression [21]. Unfortunately, the use of TZD today has been limited because of serious side effects such as fluid retention, congestive heart failure and decrease in bone mineral density [43, 44]. SR1664, a synthetic molecule that bound to PPARγ, was reported to exhibit a potent anti-diabetic activity without causing those side effects in insulin resistant mice. The effect of SR1664 was considered to be associated with an inhibition in CDK5 mediated PPARγ phosphorylation at Ser273 [45]. Similarly, in the present study, DHA was also observed to significantly block PPARγ phosphorylation and was more pronounced than EPA in stimulating adiponectin expression. Meanwhile, other previous evidences demonstrated that the modulations of lipid profiles [46], inflammation [32] and adipocytes differentiation [42] by DHA were better than EPA. All the findings suggest that a proper administration of DHA rather than EPA may provide a new approach to reduce side effects caused by TZD and DHA is more beneficial than EPA in reducing risks of NCDs.

## Conclusions

In conclusion, results of the present study demonstrated that DHA was more potent than EPA in stimulating adiponectin synthesis and secretion at relative low concentrations. Although both DHA and EPA regulated adiponectin through PPARγ and its phosphorylation, DHA led to a greater increase in PPARγ expression, and their effects on PPARγ phosphorylation were opposing: DHA inhibited PPARγ phosphorylation while EPA stimulated it. Our research is the first to indicate that the individual effects of DHA and EPA on adiponectin were partially due to their differences in regulation of PPARγ and its phosphorylation. All the findings suggested DHA may be more beneficial than EPA in the prevention and treatment of NCDs, but these should be further verified by more researches.

## Abbreviations

ANOVA: One-way analysis of variance
BSA: Bovine serum albumin
CDK5: Cyclin dependent kinase 5
DHA: Docosahexaenoic acid
DMEM: Dulbecco’s Modified Eagle’s Medium
DMSO: Dimethylsulfoxide
EPA: Eicosapentaenoic acid
IBMX: 3-isobutyl-1-methyl-xanthine
MDA: Malondialdehyde
MTT: 3-(4, 5-dimethylthiazol-2-yl)-2, 5-diphenyl tetrazolium bromide
NCDs: Non-communicable diseases
OD: Optical density
PPARγ: Peroxisome proliferator-activated receptor γ
RCTs: Randomized controlled trials
SD: Standard deviations
SDS-PAGE: Sodium dodecyl sulfate polyacrylamide gel electrophoresis
Ser: Serine
SNK: Student-Newman-Keuls
SOD: Superoxide dismutase
TBST: Tris-buffered saline-Tween
TNF-α: Tumor necrosis factor α
TZD: Thiazolidinedione
